# Impact of symptom duration and mechanical circulatory support on prognosis in cardiogenic shock complicating acute myocardial infarction

**DOI:** 10.1007/s12471-024-01881-9

**Published:** 2024-07-02

**Authors:** Florien Klein, Caïa Crooijmans, Elma J. Peters, Marcel van ’t Veer, Marijke J. C. Timmermans, José P. S. Henriques, Niels J. W. Verouden, Adriaan O. Kraaijeveld, Jeroen J. H. Bunge, Erik Lipsic, Krischan D. Sjauw, Robert-Jan M. van Geuns, Admir Dedic, Eric A. Dubois, Martijn Meuwissen, Peter Danse, Gabe Bleeker, José M. Montero-Cabezas, Irlando A. Ferreira, Jan Brouwer, Koen Teeuwen, Luuk C. Otterspoor, PCI registration committee of the Netherlands Heart Registration

**Affiliations:** 1 Heart Centre, Department of Interventional Cardiology, Catharina Hospital Eindhoven, Eindhoven, The Netherlands; 2 Heart Centre, Department of Cardiology, Amsterdam University Medical Centres, Amsterdam, The Netherlands; 3 Netherlands Heart Registration, Utrecht, The Netherlands; 4 Department of Cardiology, Utrecht University Medical Centre, Utrecht, The Netherlands; 5 Department of Cardiology, Erasmus University Medical Centre, Rotterdam, The Netherlands; 6 Department of Intensive Care, Erasmus University Medical Centre, Rotterdam, The Netherlands; 7 Department of Cardiology, University Medical Centre Groningen, Groningen, The Netherlands; 8 Department of Cardiology, St. Antonius Hospital, Nieuwegein, The Netherlands; 9 Department of Cardiology, Radboud University Medical Centre, Nijmegen, The Netherlands; 10 Department of Cardiology, Noordwest Clinics, Alkmaar, The Netherlands; 11 Department of Cardiology, Amphia Hospital, Breda, The Netherlands; 12 Department of Cardiology, Rijnstate Hospital, Arnhem, The Netherlands; 13 Department of Cardiology, Haga Hospital, The Hague, The Netherlands; 14 Department of Cardiology, Leiden University Medical Centre, Leiden, The Netherlands; 15 Department of Cardiology, Isala Hospital, Zwolle, The Netherlands; 16 Department of Cardiology, Medical Centre Leeuwarden, Leeuwarden, The Netherlands

**Keywords:** Cardiogenic shock, Acute myocardial infarction, Symptom duration, Mortality

## Abstract

**Background:**

Mortality rates in patients with cardiogenic shock complicating acute myocardial infarction (AMICS) remain high despite advancements in AMI care. Our study aimed to investigate the impact of prehospital symptom duration on the prognosis of AMICS patients and those receiving mechanical circulatory support (MCS).

**Methods and results:**

We conducted a retrospective cohort study with data registered in the Netherlands Heart Registration. A total of 1,363 patients with AMICS who underwent percutaneous coronary intervention between 2017 and 2021 were included. Patients presenting after out-of-hospital cardiac arrest were excluded. Most patients were male (68%), with a median age of 69 years (IQR 61–77), predominantly presenting with ST-elevation myocardial infarction (86%). The overall 30-day mortality was 32%. Longer prehospital symptom duration was associated with a higher 30-day mortality with the following rates: < 3 h, 26%; 3–6 h, 29%; 6–24 h, 36%; ≥ 24 h, 46%; *p* < 0.001. In a subpopulation of AMICS patients with MCS (*n* = 332, 24%), symptom duration of > 24 h was associated with significantly higher mortality compared to symptom duration of < 24 h (59% vs 45%, *p* = 0.029). Multivariate analysis identified > 24 h symptom duration, age and in-hospital cardiac arrest as predictors of 30-day mortality in MCS patients.

**Conclusion:**

Prolonged prehospital symptom duration was associated with significantly increased 30-day mortality in patients presenting with AMICS. In AMICS patients treated with MCS, a symptom duration of > 24 h was an independent predictor of poor survival. These results emphasise the critical role of early recognition and intervention in the prognosis of AMICS patients.

**Supplementary Information:**

The online version of this article (10.1007/s12471-024-01881-9) contains supplementary material, which is available to authorized users.

## What’s new?

- Following the exclusion of those with out-of-hospital cardiac arrest, the observed 30-day mortality of patients with cardiogenic shock complicating acute myocardial infarction (AMICS) who underwent percutaneous coronary intervention was 32%.
- Prolonged prehospital symptom duration was associated with significantly increased 30-day mortality in AMICS patients.
- In 24% of all AMICS patients mechanical circulatory support (MCS) was used.
- In AMICS patients with MCS, symptom duration > 24 h was an independent predictor of 30-day mortality.

## Introduction

Cardiogenic shock (CS) is a life-threatening condition caused by severe cardiac dysfunction, leading to hypotension and organ hypoperfusion, resulting in end-organ failure that is often followed by death. Clinical manifestations of CS include hypotension, signs of organ hypoperfusion (e.g. decreased urine output, altered mental status) and peripheral vasoconstriction. Biochemical manifestations of CS include metabolic acidosis, and elevated lactate and creatinine levels [1–3].

Acute myocardial infarction (AMI) is the most common cause of CS [2, 4]. Coronary obstruction during AMI impairs myocardial perfusion, resulting in ischaemia-driven myocardial necrosis and subsequent ventricular dysfunction. In CS complicating AMI (AMICS) this induces a vicious cycle in which regional myocyte loss reduces cardiac output, inducing further coronary ischaemia, eventually leading to irreversible tissue loss, and often deteriorating further until death. Recently the influence of a simultaneously developing systemic reaction has been acknowledged, whereby microcirculatory dysfunction and systemic inflammation further contribute to the worsening of shock. This underlines that early recognition and treatment are crucial for prognosis [2, 5].

Advancements in diagnosis and treatment of AMI have led to a decrease in the incidence of AMICS, which currently complicates 4–12% of AMI cases [1]. The implementation of early revascularisation strategies following the publication of the SHOCK trial in 1999 resulted in lower mortality rates [6, 7]. However, no other interventions, not even the emergence of mechanical circulatory support (MCS), have since proved to have a beneficial effect on survival, and mortality remains high at 35- to 50% [2, 3, 5, 8–11].

As early recognition and treatment are undoubtedly crucial for prognosis, better insights are required regarding the impact of symptom duration in patients with AMICS. Therefore, we aimed to determine the association between symptom duration and outcomes in AMICS patients, as well as in a subgroup of these patients who received MCS.

## Materials and methods

### Study design and eligibility

We conducted a retrospective, multicentre study analysing data from 14 Dutch heart centres registered in the Netherlands Heart Registration (NHR). The NHR is a nationwide quality registry that contains procedural and outcome data on all invasive cardiac procedures from Dutch hospitals [12]. Patients with CS undergoing percutaneous coronary intervention (PCI) for AMI between January 2017 and September 2021 were identified, and predefined variables were collected. Participating hospitals and investigators are listed in Table S1 (Electronic Supplementary Material). For this study, patients presenting after an out-of-hospital cardiac arrest (OHCA) were excluded.

CS was defined as the presence of hypotension along with signs of end-organ hypoperfusion before, during or after leaving the catheterisation laboratory. Criteria for hypotension were systolic blood pressure ≤ 90 mm Hg for 30 min or the need for therapy (infusion, inotropic drugs or mechanical assist device) to maintain blood pressure > 90 mm Hg. Signs of end-organ hypoperfusion consist of cold extremities, oliguria (< 30 ml/h) or heart rate ≥ 60 bpm.

PCI was defined as any intervention in which an instrument (guide wire, balloon, thrombosuction catheter, rotablation etc.) is introduced into one of the coronary arteries or into the coronary artery bypass graft with the intention of treating the affected vessel.

### Study endpoints

The primary endpoint of this study was 30-day mortality according to symptom duration group. Additionally, we assessed the impact of symptom duration on 30-day mortality in AMICS patients with MCS. Furthermore, we aimed to identify additional predictors of 30-day mortality in AMICS patients with MCS. Secondary endpoints include characteristics of patients with longer symptom duration and of patients with MCS utilisation.

### Data collection

Cardiogenic shock variables were established after a consensus was reached by interventional cardiologists and intensive care physicians from the participating hospitals. Peters et al. present a detailed description of this process [13]. Symptom duration before hospital presentation was retrieved from the electronic patient file and subdivided into four groups: < 3 h, 3–6 h, 6–24 h and > 24 h. Survival status was retrieved from the electronic patient file or the governmental personal records database (Dutch: *Basisregistratie Personen*) in all hospitals. Duplicates were identified, defined as PCI performed within a time frame of 100 days. To prevent inconsistency of data, we included the initial registration for each patient.

### Statistical analysis

Categorical data are presented as number of patients or proportions with corresponding percentages. All continuous variables had non-normal distribution and are therefore presented as medians with interquartile range (IQR). Differences in characteristics were assessed using chi-square tests or Fisher’s exact test for categorical variables, and Kruskal-Wallis or Mann-Whitney‑U test for continuous variables. Patient characteristics were compared between subgroups stratified by symptom duration and MCS use. Survival rates stratified for symptom duration were calculated using the Kaplan-Meier method, with the log-rank test for group comparison. Logistic regression analysis was performed to identify predictors of 30-day mortality in the MCS population. Due to the small number of patients in this subgroup, symptom duration was dichotomised at 24 h. Variables considered relevant or that demonstrated a significant association with mortality in univariate analysis (*p* < 0.10) were included in the multivariate analysis. Results are displayed as odds ratio with 95% confidence interval. Two-tailed tests were applied to assess significance, with a *p*-value of < 0.05 considered statistically significant. All statistical analyses were performed using SPSS Statistics, version 28.0.1.1 (IBM Corp., Armonk, NY, USA).

## Results

### CS population

**Fig. 1:**
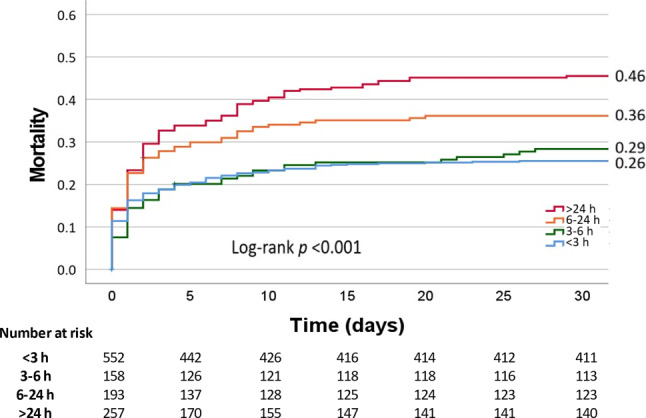
Thirty-day mortality stratified by symptom duration in patients with cardiogenic shock complicating acute myocardial infarction

From January 2017 to September 2021, data from 2,328 patients was collected. After exclusion of duplicates (*n* = 21) and OHCA patients (*n* = 944), 1,363 patients remained. Among all participants, 68% were male, with a median age of 69 (IQR 61–77) years. Patients with longer symptom duration were more frequently diagnosed with non-ST-elevation myocardial infarction (NSTEMI), were more likely to have diabetes mellitus, and received vasoactive agents and MCS more often (Tab. 1). Notably, 17% of all patients underwent PCI involving two or more vessels. Multivessel PCI was associated with higher 30-day mortality (PCI of 1 vs ≥ 2 vessels: 30% vs 44%, *p* < 0.001). The overall 30-day mortality was 32%, and patients with longer symptom duration showed significantly higher mortality rates: < 3 h, 26%; 3–6 h, 29%; 6–24 h, 36%; ≥ 24 h, 46%, *p* < 0.001 (Fig. 1).

**Table 1:** Characteristics of the patients with cardiogenic shock complicating acute myocardial infarction stratified according to symptom duration (hours)

	CS population	< 3 h	3–6 h	6–24 h	> 24 h	*p*-value
*Patient characteristics*						
Male	789/1,167 (68)	394/554 (71)	100/160 (63)	121/195 (62)	174/258 (67)	0.051
Age (years)	69 (61–77)	69 (59–77)	71 (62–77)	71 (62–79)	69 (62–76)	0.208
BMI (kg/cm^2^)	26 (24–29)	26 (24–29)	26 (24–29)	26 (23–29)	26 (24–29)	0.984
Indication PCI:						< 0.001
– STEMI	964/1,126 (86)	504/536 (94)	146/159 (92)	155/194 (80)	159/237 (67)	
– NSTEMI	162/1,126 (14)	32/536 (6)	13/159 (8)	39/194 (20)	78/237 (33)	
Diabetes	256/1,138 (23)	98/541 (18)	31/155 (20)	53/191 (28)	74/251 (30)	< 0.001
Multivessel disease	761/1,159 (66)	333/551 (60)	107/158 (68)	137/193 (71)	184/257 (72)	0.004
In-hospital cardiac arrest	105/1,163 (9)	60/554 (11)	9/159 (6)	11/193 (6)	25/257 (10)	0.067
Vasoactive agents pre-PCI:	338/1,167 (29)	127/554 (23)	45/160 (28)	55/195 (28)	111/258 (43)	< 0.001
– 1	189/338 (56)	84/127 (66)	25/45 (56)	28/55 (51)	52/111 (47)	
– 2	115/338 (34)	35/127 (28)	17/45 (38)	23/55 (42)	40/111 (36)	
– ≥ 3	34/338 (10)	8/127 (6)	3/45 (7)	4/55 (7)	19/111 (17)	
*Haemodynamics on admission*						
Systolic blood pressure (mm Hg)	97 (80–118)	99 (80–120)	90 (75–142)	100 (82–119)	96 (80–117)	0.051
Diastolic blood pressure (mm Hg)	60 (48–74)	60 (47–75)	55 (43–69)	62 (50–76)	60 (50–70)	0.053
MAP (mm Hg)	73 (59–88)	73 (58–90)	67 (55–85)	75 (61–90)	73 (60–86)	0.039
Heart rate (bpm)	78 (59–100)	73 (55–90)	75 (53–98)	84 (66–107)	89 (64–110)	< 0.001
*Laboratory results on admission*						
Lactate (mmol/l)	3.7 (2.0–6.6)	3.7 (2.0–6.6)	2.9 (2.0–6.3)	4.5 (2.3–7.3)	3.4 (1.9–6.3)	0.229
Creatinine (µmol/l)	96 (79–126)	92 (77–115)	95 (80–125)	95 (77–132)	106 (85–153)	< 0.001
Haemoglobin (mmol/l)	8 (7–9)	8.4 (7.5–9.2)	8.0 (7.2–8.8)	8.1 (7.2–9.1)	7.5 (6.5–8.6)	< 0.001
Peak hs-Tn‑T (ng/l)^a^	4,350 (1,136–10,000)	4,540 (896–10,076)	3,155 (902–10,000)	5,494 (1,510–10,000)	3,873 (1,614–10,000)	0.232
Peak CK-MB (U/l)^a^	188 (62–465)	188 (65–503)	247 (83–489)	231 (70–590)	138 (39–341)	0.024
*Angiographic features*						
First treated vessel:						0.018
– Left main artery	150/1,062 (14)	64/505 (13)	14/149 (9)	30/172 (17)	42/236 (18)	
– Left anterior descending artery	342/1,062 (32)	165/505 (33)	49/149 (33)	56/172 (33)	72/236 (31)	
– Circumflex artery	144/1,062 (14)	55/505 (11)	21/149 (14)	26/172 (15)	42/236 (18)	
– Right coronary artery	411/1,062 (39)	215/505 (43)	62/149 (42)	60/172 (35)	74/236 (31)	
– Venous or arterial graft	15/1,062 (1)	6/505 (1)	3/149 (2)	0/172 (0)	6/236 (3)	
≥ 2 vessels treated	193/1,160 (17)	72/554 (13)	33/160 (21)	35/195 (18)	54/258 (21)	0.013
TIMI flow before PCI:						< 0.001
– 0/1	771/979 (79)	397/473 (84)	111/139 (80)	127/164 (77)	136/203 (67)	
– 2	99/979 (10)	43/473 (9)	12/139 (9)	18/164 (11)	26/203 (13)	
– 3	109/979 (11)	33/473 (7)	16/139 (12)	19/164 (12)	41/203 (20)	
TIMI flow after PCI:						< 0.001
– 0/1	94/1,000 (9)	33/394 (7)	10/141 (7)	20/163 (12)	31/210 (12)	
– 2	119/1,000 (12)	59/394 (12)	12/141 (9)	26/163 (16)	22/210 (9)	
– 3	787/1,000 (79)	394/394 (81)	119/141 (84)	117/163 (72)	157/210 (61)	
MCS	281/1,159 (24)	104/548 (19)	44/159 (28)	57/194 (29)	76/258 (30)	0.001
*Outcome*						
Thirty-day mortality	373/1,160 (32)	141/552 (26)	45/158 (29)	70/193 (36)	117/257 (46)	< 0.001
One-year mortality	321/826 (39)	117/394 (30)	36/108 (33)	64/139 (46)	104/185 (56)	< 0.001

Nominal data are presented as *n* (%), continuous data as median (IQR)

*CS* cardiogenic shock, *BMI* body mass index, *PCI* percutaneous coronary intervention, *NSTEMI* non-ST-elevation myocardial infarction, *STEMI* ST-elevation myocardial infarction, *Vasoactive agents pre PCI* Number of drugs administered before PCI: from noradrenaline, adrenaline, dopamine, dobutamine and enoximone/milrinone, *MAP* mean arterial pressure, *hs-Tn‑T* high-sensitivity troponin T, *CK-MB* creatine kinase-myocardial band, *TIMI* thrombolysis in myocardial infarction, *MCS* mechanical circulatory support, *IQR* interquartile range

^a^Peak values within 3 days after PCI

### MCS versus non-MCS population

In 332 patients (24%), MCS was used. The distribution of MCS types can be found in Table S2 (Electronic Supplementary Material).

**Fig. 2:**
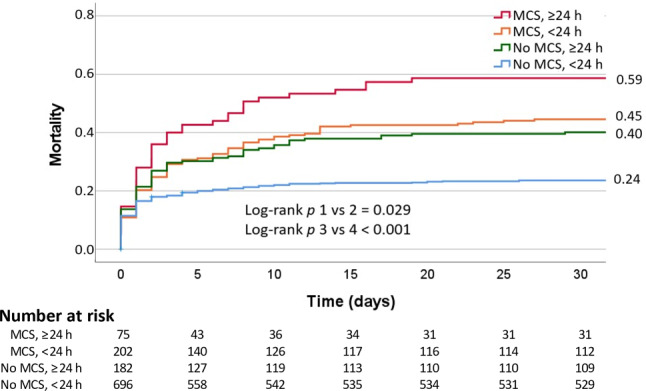
Thirty-day mortality stratified by symptom duration and use of mechanical circulatory support (*MCS*)

Patients with MCS presented with prolonged symptom duration, higher heart rates, and vasoactive medication was administered more often, compared with non-MCS patients (Tab. 2). Moreover, observed levels of lactate (4.6 vs 3.2 mmol/l, *p* < 0.001), troponin (10,000 vs 3,318 ng/l, *p* < 0.001) and creatine kinase—myocardial band (CK-MB; 347 vs 129 U/l, *p* < 0.001) were significantly higher in the MCS population. Also, multivessel disease was more often present (77% vs 63%, *p* < 0.001), and PCI of the left main coronary artery was performed more often in this subgroup (24% vs 12%, *p* < 0.001). Mortality at 30 days was significantly higher in the MCS population (50% vs 29%, *p* < 0.001). Patients who experienced a symptom duration of more than 24 h, with or without MCS use, had notably higher mortality rates, as demonstrated in Fig. 2.

**Table 2:** Comparison of study groups stratified by use of mechanical circulatory support (*MCS*)

	MCS	Non-MCS	*p*-value
*Patient characteristics*			
Male	231/332 (70)	672/1,016 (66)	0.248
Age (years)	69 (60–75)	70 (62–78)	< 0.001
BMI (kg/cm^2^)	26 (24–29)	26 (24–29)	0.698
Diabetes	83/320 (26)	216/994 (22)	0.118
Indication PCI			0.845
– NSTEMI	67/327 (20)	194/1,012 (19)	
– STEMI	260/327 (80)	818/1,012 (81)	
In-hospital cardiac arrest	36/331 (11)	97/1,011 (10)	0.498
Onset AMI symptoms (hours)			0.001
– < 3 h	104/281 (37)	444/878 (51)	
– 3–6 h	44/281 (16)	115/878 (13)	
– 6–24 h	57/281 (20)	137/878 (16)	
– > 24 h	76/281 (27)	182/878 (21)	
Vasoactive agents pre-PCI	126/332 (38)	277/1016 (27)	< 0.001
Number of vasoactive agents pre-PCI			< 0.001
– 1	53/126 (42)	170/277 (61)	
– 2	49/126 (39)	86/277 (31)	
– ≥ 3	24/126 (19)	21/277 (8)	
*Haemodynamics on admission*			
Systolic blood pressure (mm Hg)	95 (78–118)	97 (80–119)	0.497
Diastolic blood pressure (mm Hg)	60 (47–74)	60 (47–73)	0.480
MAP (mm Hg)	72 (57–87)	73 (59–89)	0.422
Heart rate (bpm)	90 (70–110)	75 (56–95)	< 0.001
*Laboratory values on admission*			
Lactate (mmol/l)	4.6 (2.5–7.2)	3.2 (1.8–6.2)	< 0.001
Creatinine (µmol/l)	100 (82–134)	96 (78–126)	0.27
Haemoglobin (mmol/l)	8.1 ± 1.5	8.0 ± 1.4	0.161
Glucose (mmol/l)	11.3 (8.9–16.3)	9.7 (7.8–13.2)	< 0.001
Peak hs-Tn‑T (ng/l^a^)	10,000 (2,360–21,401)	3,318 (929–8,919)	< 0.001
Peak CK-MB (U/l^a^)	347 (138–695)	129 (46–580)	< 0.001
*Angiographic features*			
Multivessel disease	254/331 (77)	631/1,008 (63)	< 0.001
CTO	10/332 (3)	14/1,016 (1)	0.051
First treated vessel			< 0.001
– Left main artery	69/292 (24)	111/930 (12)	
– Left anterior descending artery	103/292 (35)	296/930 (32)	
– Circumflex artery	42/292 (14)	121/930 (13)	
– Right coronary artery	74/292 (25)	388/930 (42)	
– Venous or arterial graft	4/292 (1)	14/930 (2)	
*Outcome*			
Thirty-day mortality	165/328 (50)	290/1,012 (29)	< 0.001
One-year mortality	128/256 (50)	260/707 (37)	< 0.001

Nominal data are presented as *n* (%), continuous data as median (IQR)

*BMI* body mass index, *PCI* percutaneous coronary intervention, *NSTEMI* non-ST-elevation myocardial infarction, *STEMI* ST-elevation myocardial infarction, *AMI* acute myocardial infarction, *Vasoactive agents pre-PCI* Number of drugs administered before PCI: from noradrenaline, adrenaline, dopamine, dobutamine and enoximone/milrinone, *MAP* mean arterial pressure, *hs-Tn‑T* high-sensitivity troponin T, *CK-MB* creatine kinase-myocardial band, *CTO* chronic total occlusion, *IQR* interquartile range

^a^Peak values within 3 days after PCI

No significant correlation was found between symptom duration (less or more than 24 h) and the selected MCS device, although notable trends emerged. Extracorporeal membrane oxygenation device, either alone or in combination with Impella (Abiomed, Danvers, MA, USA) or intra-aortic balloon pump (IABP), demonstrated a tendency toward higher usage in patients with a symptom duration > 24 h (17% vs 25%, *p* = 0.185). Conversely, IABP usage was lower among patients presenting with symptoms lasting more versus less than 24 h (51% vs 60%, *p* = 0.341; (Table S2, Electronic Supplementary Material)). IABP usage after 24‑h symptom duration demonstrated a trend towards significance in association with higher mortality (55% vs 38%, *p* = 0.061; Table S3, Electronic Supplementary Material).

Due to the significant amount of missing data, some variables (e.g. haemodynamic parameters, laboratory results and thrombolysis in myocardial infarction (TIMI) flow post-PCI) were excluded from the multivariate analysis. Multivariate analysis (Tab. 3) indicated three significant predictors of mortality in AMICS patients treated with MCS, including age, in-hospital cardiac arrest and symptom duration > 24 h.

**Table 3:** Univariate and multivariate predictors of 30-day mortality in patients with cardiogenic shock complicating acute myocardial infarction receiving mechanical circulatory support (*MCS*)

	Univariate analysis			Multivariate analysis		
Variable	Odds ratio	95% CI	p‑value	Odds ratio	95% CI	p‑value
Male sex	1.05	0.66–1.67	0.847			
Age	1.02	1.00–1.04	0.038	1.03	1.01–1.06	0.010
Multivessel disease	2.06	1.21–3.51	0.008	1.59	0.77–3.29	0.210
Diabetes	1.49	0.90–2.47	0.125			
IHCA	2.13	1.03–4.42	0.043	3.68	1.34–10.07	0.011
Symptom duration > 24 h	1.77	1.03–3.02	0.038	2.32	1.21–4.45	0.011
Vasoactive agents	1.38	0.88–2.16	0.162			
Vasoactive agents ≥ 2	1.27	0.75–2.15	0.379			
PCI indication NSTEMI	1.69	0.92–3.01	0.088	0.80	0.36–1.78	0.582
Left main target vessel	1.28	0.75–2.21	0.369	0.99	0.51–1.91	0.974
PCI of ≥ 2 vessels	0.66	0.40–1.07	0.656	0.95	0.49–1.82	0.873
TIMI flow after PCI < 3	1.71	0.96–3.03	0.067			
Timing MCS pre-PCI	1.48	0.94–2.34	0.092	0.939	0.51–1.72	0.837
*Haemodynamics*						
Heart rate	1.01	1.00–1.02	0.009			
Systolic blood pressure	0.98	0.98–0.99	< 0.001			
Diastolic blood pressure	0.98	0.97–1.00	0.006			
MAP	0.98	0.97–0.99	0.001			
*Laboratory results*						
Lactate	1.13	1.05–1.21	0.001			
Glucose	1.06	1.01–1.10	0.008			
Creatinine	1.01	1.00–1.01	0.002			
CK-MB	1.00	1.00–1.00	0.376			
Troponin	1.00	1.00–1.00	0.172			

*CI* confidence interval, *IHCA* in-hospital cardiac arrest, *PCI* percutaneous coronary intervention, *NSTEMI* non-ST-elevation myocardial infarction, *TIMI* thrombolysis in myocardial infarction, *MAP* mean arterial pressure, *CK-MB* creatine kinase-myocardial band

## Discussion

This study examined the impact of symptom duration on prognosis in AMICS patients undergoing PCI. Prolonged symptom duration before hospital presentation was significantly associated with increased 30-day mortality. In the population treated with MCS, symptom duration > 24 h was also associated with higher mortality and was an independent predictor in a multivariate analysis for 30-day mortality.

With over 1,300 patients included, our study provides real-world data on AMICS care in the Netherlands. Our observed 30-day mortality falls within the lower range of rates documented in previous studies [1, 2, 5, 14, 15]. We observed a significant increase in 30-day mortality as prehospital symptom duration prolonged, consistent with the subgroup analysis conducted in the SHOCK trial [6]. In this trial, early revascularisation did not lead to a significant reduction in the primary endpoint, 30-day mortality. However, a subgroup analysis focusing on patients randomised within 6 h of symptom onset (approximately one quarter of the study population, *n* = 73) did reveal a significant decrease in 30-day mortality [16]. Furthermore, the long-term evaluation in the SHOCK trial showed higher 1‑year mortality rates (although not significant) associated with increasing time intervals from myocardial infarction to revascularisation, ranging from 0 to 8 h (< 4 h, 36%; 4 to < 6 h, 55%; 6 to < 8 h, 82%) [17].

We excluded patients with OHCA to enhance the homogeneity of our study cohort. It is noteworthy that mortality in these excluded patients differed significantly from that in our non-OHCA AMICS population (47% vs 34%, *p* < 0.001). In the OHCA population, no significant correlation was identified between symptom duration and mortality. OHCA patients constitute a distinctive subgroup within AMICS, often present within a short time frame with severe shock, due to cardiac dysfunction and systemic effects of whole-body ischaemia-reperfusion injury [18]. Mortality is high and often driven by anoxic brain injury and multiorgan failure, a condition that limits the potential for improving prognosis through interventions such as MCS, even if myocardial recovery is successful. Important prehospital prognostic factors for OHCA patients include time to first cardiopulmonary resuscitation and time until return of spontaneous circulation [19, 20], aspects that have not been included in our assessment. By excluding OHCA patients, we aimed to provide accurate outcomes of AMICS regarding symptom duration.

In our subgroup analysis, we compared patients receiving MCS with those who did not. Within the MCS group, vasoactive agents were administered more frequently, heart rates were higher, and higher levels of lactate, troponin and CK-MB were observed, indicating substantial disparity in shock severity. Patients receiving MCS appeared to be in a more critical condition, necessitating support. Consequently, the use of MCS in our study is likely biased towards those with more advanced shock severity, impacting the observed mortality differences.

No significant correlation was found between symptom duration and the MCS device selected. However, a trend towards significance emerged concerning patients receiving IABP after 24 h, demonstrating higher mortality rates compared to initiation before 24 h (55% vs 38%, *p* = 0.061). These findings support the view that IABP, considered a less potent device, might be less effective in later stages of CS [21], emphasising the importance of a personalised MCS strategy, tailored to individual patient characteristics.

Comparing our MCS subgroup with the IABP group in the IABP-SHOCK II trial [22], our mortality rates are higher (50% vs 40%). This finding might be a result of selective patient enrollment, as the IAPB-SHOCK II trial excluded patients with onset of shock > 12 h, potentially having a favourable effect on outcomes.

The mortality observed in our MCS population corresponds to those reported in the recently published ECLS-SHOCK trial (50% vs 48%) [11]. Notably, this trial did not exclude OHCA patients, who constituted 78% of the ECLS population. In contrast to our study, subanalysis within the ECLS cohort revealed no significant mortality differences between patients presenting with or without OHCA.

Multivariate logistic regression analysis identified > 24 h symptom duration as an independent predictor for 30-day mortality in the AMICS population receiving MCS. As the duration of shock increases, a cascade of progressive systemic inflammation and multiorgan failure is initiated. Once cardiometabolic shock is established, therapeutic interventions may fail to reverse the downward spiral and improve survival. Hence, early shock recognition and treatment, including timely usage of MCS, might improve prognosis. In practical terms, this necessitates early referral and accurate recognition of shock, which could shorten the time to MCS implantation and may enhance survival. Furthermore, patient selection could impact the effectiveness of MCS regarding clinical outcomes. In cases of severe cardiometabolic shock, therapeutic interventions have a limited impact on prognosis, whereas patients with deteriorating early-stage shock might derive greater benefit from timely intervention. Additionally, as previously discussed, a personalised MCS strategy could potentially enhance prognosis.

There are some important potential limitations associated with our study. Firstly, the retrospective design of this study makes it more susceptible to selection bias, impacting the reliability of our findings. Secondly, missing data could have hindered identifying significant prognostic variables, particularly within the MCS population where some variables (e.g. haemodynamic parameters, laboratory results and TIMI flow post-PCI) were excluded from the multivariate analysis. Variables with respective percentages of missing data are provided in Supplementary Table S4 (Electronic Supplementary Material). Thirdly, 17% of the population underwent multivessel PCI, which was associated with higher 30-day mortality rates and contradicts the established standard of care highlighted by the CULPRIT-SHOCK trial [23].

## Conclusion

Prolongation of prehospital symptom duration is associated with significantly increased 30-day mortality in AMICS patients without OHCA. In patients treated with MCS, symptom duration > 24 h significantly increased 30-day mortality. These results emphasise the critical role of early recognition and intervention in AMICS. Further prospective studies are needed to confirm if early timing of MCS could improve the outcome in this group.

### Supplementary Information

Table S1 Participating centers and physicians

Table S2 Types of mechanical circulatory support device and distribution stratified for symptom duration.

Table S3 30-day mortality stratified by type of MCS device and symptom duration

Table S4 Presented variables with missing data
