# Supplementary material for: Impact of symptom duration and mechanical circulatory support on prognosis in cardiogenic shock complicating acute myocardial infarction
Source: Neth Heart J. 2024 Jul 2;32(7-8):290–7. doi: 10.1007/s12471-024-01881-9 (PMC11239615; doi:10.1007/s12471-024-01881-9)
Supplement: Supplementary file 1 — Table S1 Participating centers and physicians [file 12471_2024_1881_MOESM1_ESM.docx]

**Table S1**

| **Participating centers and physicians** | |
| --- | --- |
| **Hospital** | **Physician** |
| Amphia Ziekenhuis | Dr. M. Meuwissen |
| Amsterdam Universitair Medische Centra, AMC | Prof. dr. J.P. Henriques |
| Amsterdam Universitair Medische Centra, VU | Dr. C.J.W. Verouden |
| Catharina Ziekenhuis | Dr. L.C. Otterspoor  Dr. K. Teeuwen |
| Erasmus Medisch Centrum | Drs. J.J.H. Bunge  Dr. E.A. Dubois |
| HagaZiekenhuis | Dr. G.B. Bleeker |
| Isala ziekenhuis | Dr. I. Andrade Ferreira |
| Leids Universitair Medisch Centrum | Drs. J. Montero-Cabezas |
| Sint Antonius Ziekenhuis | Dr. K.D. Sjauw |
| Noordwest Ziekenhuisgroep | Drs. A. Dedic |
| Radboud Universitair Medisch Centrum | Prof. dr. R.J. van Geuns |
| Rijnstate Ziekenhuis | Dr. P.W. Danse |
| Universitair Medisch Centrum Groningen | Dr. E. Lipšic |
| Universitair Medisch Centrum Utrecht | Dr. A.O. Kraaijeveld |
