# Supplementary material for: Impact of symptom duration and mechanical circulatory support on prognosis in cardiogenic shock complicating acute myocardial infarction
Source: Neth Heart J. 2024 Jul 2;32(7-8):290–7. doi: 10.1007/s12471-024-01881-9 (PMC11239615; doi:10.1007/s12471-024-01881-9)
Supplement: Supplementary file 2 — Table S2 Types of mechanical circulatory support device and distribution stratified for symptom duration. [file 12471_2024_1881_MOESM2_ESM.docx]

**Table S2**: Types of mechanical circulatory support device and distribution stratified for symptom duration.

|  | **Total MCS population** | **<24h symptom duration** | **>24h symptom duration** | **p-value** |
| --- | --- | --- | --- | --- |
| **MCS** |  |  |  |  |
| IABP | 183/332 (55) | 122/205 (60) | 39/76 (51) | 0.341 |
| Transvalvular axial flow device | 66/332 (20) | 44/205 (22) | 14//76 (18) | 0.424 |
| ECMO binned (with or without impella/IABP) | 75/332 (23) | 35/205 (17) | 19/76 (25) | 0.185 |
| IABP + Transvalvular axial flow device _a_ | 3 /332 (1) | 2/205 (1) | 1/76 (1) | 1.000 |
| Other | 5/332 (2) | 2/205 (1) | 3/76 (4) | 1.000 |
| Presented as number with corresponding percentage.  MCS = mechanical circulatory support, IABP = intraaortic balloon pump, ECMO = extracorporeal membrane oxygenation  α impella® | |  |  |  |
