# Supplementary material for: Impact of symptom duration and mechanical circulatory support on prognosis in cardiogenic shock complicating acute myocardial infarction
Source: Neth Heart J. 2024 Jul 2;32(7-8):290–7. doi: 10.1007/s12471-024-01881-9 (PMC11239615; doi:10.1007/s12471-024-01881-9)
Supplement: Supplementary file 3 — Table S3 30-day mortality stratified by type of MCS device and symptom duration [file 12471_2024_1881_MOESM3_ESM.docx]

**Table S3:** 30-day mortality stratified by type of MCS device and symptom duration

| **Mortality** | **30-day mortality** | **p-value** |
| --- | --- | --- |
| **IABP** |  | 0.061 |
| <24h symptom duration | 45/120 (38) |  |
| >24h symptom duration | 21/38 (55) |  |
| **Transvalvular axial flow device** |  | 0.940 |
| <24h symptom duration | 21/43 (49) |  |
| >24h symptom duration | 7/14 (50) |  |
| **ECMO (+ other device)** |  | 0.771 |
| <24h symptom duration | 22/35 (63) |  |
| >24h symptom duration | 13/19 (68) |  |
| **IABP + Transvalvular axial flow device** _a_ |  | 1.000 |
| <24h symptom duration | 1/2 (50) |  |
| >24h symptom duration | 1/1 (100) |  |
| **Other** |  | 1.000 |
| <24h symptom duration | 1/2 (50) |  |
| >24h symptom duration | 2/3 (67) |  |
| Presented as number with corresponding percentage.  MCS = mechanical circulatory support, IABP = intraaortic balloon pump, ECMO = extracorporeal membrane oxygenation  α impella® | | |
