# Supplementary material for: Impact of symptom duration and mechanical circulatory support on prognosis in cardiogenic shock complicating acute myocardial infarction
Source: Neth Heart J. 2024 Jul 2;32(7-8):290–7. doi: 10.1007/s12471-024-01881-9 (PMC11239615; doi:10.1007/s12471-024-01881-9)
Supplement: Supplementary file 4 — Table S4 Presented variables with missing data [file 12471_2024_1881_MOESM4_ESM.docx]

**Table S4:** Presented variables with missing data

| **Variable** | | **Outcome** | | **Missing No. (%)** |
| --- | --- | --- | --- | --- |
| **Age** *Difference between date of birth and date of intervention.* | | Continuous | | 0 (0) |
| **Sex** | | Male Female | | 0 (0) |
| **Diabetes mellitus** *Indicate the most intensive therapy that was used to treat diabetes* | | None Diabetes, treatment unknown Diabetes, no treatment Diabetes, diet Diabetes, oral medication Diabetes, insulin Diabetes, other | | 34 (2.5) |
| **Multivessel disease** *Presence of multivessel disease during the current intervention. For first interventions: stenosis of ≥70% in ≥2 native vessels with a diameter of at least 1.5 mm. In patients with a prior coronary intervention: ≥70% stenosis in ≥1 native coronary arteries that have not yet been treated and/or multivessel disease during previous intervention.* | | No Yes | | 9 (0.7) |
| **Indication of PCI** *Status of the patient during the current intervention:* *NSTEMI: presence of acute chest pain in the absence of ST elevation (including stable angina);* *STEMI: presence of acute chest pain and (>20 mm) ST elevation.* | | NSTEMI STEMI | | 57 (4.2) |
| **First PCI-treated vessel** *Name of dilated coronary artery:* *LM: left main* *LAD: left coronary artery* *RCX: circumflex artery* *AL/IM: anterolateral / intermediate branch* *RCA: right coronary artery* *Venous graft* *Arterial graft* | | LM LAD RCX AL/IM RCA Venous graft Arterial graft | | 119 (8.7) |
| **Survival status** *Survival status (as determined after verification of the personal records database or date of last contact).* | | Alive Deceased | | 4 (0.3) |
| **Date of survival status** *Days between PCI and either verification of survival status (alive patients) or date of death (deceased patients).* | | Continuous | | 3 (0.2) |
| **Duration of symptoms** *Amount of time between start of symptoms and hospital presentation.* | | >24 h >12 h, ≤24 h >6 h, ≤12 h >3 h, ≤6 h ≤3 h | | 196 (14.4) |
| **Systolic blood pressure—mmHg** *Systolic blood pressure according to first in-hospital measurement pre-PCI. In case of absence of an in-hospital measurement, a measurement by the emergency medical team can be used.* | Continuous (0–300) | | 139 (10.2) | |
| **Diastolic blood pressure—mmHg** *Diastolic blood pressure according to first in-hospital measurement pre-PCI. In case of absence of an in-hospital measurement, a measurement by the emergency medical team can be used.* | Continuous (0–300) | | 160 (11.7) | |
| **Mean arterial pressure —mmHg**  *Mean arterial pressure according to first in-hospital measurement pre-PCI. In case of absence of an in-hospital measurement, a measurement by the emergency medical team can be used.* | Continuous (0–300) | | 161 (12.1) | |
| **Heart rate—bpm** *Heart rate according to first in-hospital measurement pre-PCI. In case of absence of an in-hospital measurement, a measurement by the emergency medical team can be used.* | Continuous (0–300) | | 168 (12.3) | |
| **IHCA** *Patient was defibrillated (and received chest compressions) in the hospital before entering the cath lab.* | No Yes | | 12 (0.9) | |
| **Height—kg** *Most recently reported height (measured during index admission). When height is not measured during index admission, the most recently reported height (up to one year old) can be used.* | Continuous (20–270) | | 223 (16.4) | |
| **Weight—cm** *Most recently reported weight (measured during index admission). When weight is not measured during index admission, the most recently reported weight (up to one year old) can be used.* | Continuous (0.3–250) | | 185 (13.6) | |
| **Lactate on admission—mmol/L** *First measured blood lactate level on admission (±1 h around PCI).* | Continuous (0.0–40.0) | | 657 (48.2) | |
| **Hemoglobin on admission—mmol/L** *First measured hemoglobin level on admission (±1 h around PCI).* | Continuous (0.0–15.0) | | 108 (7.9) | |
| **Glucose on admission— mmol/L** *First measured glucose level on admission (±1 h around PCI).* | Continuous (1.0–40.0) | | 221 (16.2) | |
| **Creatinine on admission—µmol/L** *First measured creatinine level on admission (±1 h around PCI).* | Continuous (1.0–2000.0) | | 164 (12.0) | |
| **CK-MB max—U/L** *Highest creatinine kinase-MB level during index admission (up to 3 days after PCI).* | Continuous (0–10,000) | | 765 (56.1) | |
| **hs-Troponin-T—µg/L** *Highest high-sensitive troponin-T level during index admission (up to 3 days after PCI).* | Continuous (0–150,000) | | 265 (19.4) | |
| **Mechanical circulatory support** *Type of mechanical circulatory support that was initiated during index admission.* | None IABP Impella ECMO IABP + ECMO Impella + ECMO IABP + Impella Other | | 15 (1.1) | |
| **Implementation Mechanical circulatory support**  *Timing of implementation of mechanical circulatory support..* | Pre-PCI  Post-PCI | | 2 (0.6) | |
| **TIMI flow grade pre-PCI** *TIMI flow measured pre-PCI.* | 0 1 2 3 | | 234 (17.2) | |
| **TIMI flow grade post-PCI** *TIMI flow measured post-PCI.* | 0 1 2 3 | | 205 (16.6) | |
| **Norepinephrine prior to PCI** *Whether or not a patient received norepinephrine pre-PCI (up to first pressure registration).* | No Yes | | 22 (1.6) | |
| **Dobutamine prior to PCI** *Whether or not a patient received dobutamine pre-PCI (up to first pressure registration).* | No Yes | | 18 (1.3) | |
| **Enoximone or milrinone prior to PCI** *Whether or not a patient received enoximone/milrinone pre-PCI (up to first pressure registration).* | No Yes | | 19 (1.4) | |
| **Adrenaline prior to PCI** *Whether or not a patient received adrenaline pre-PCI (up to first pressure registration).* | No Yes | | 30 (2.2) | |
| **Dopamine prior to PCI** *Whether or not a patient received dopamine pre-PCI (up to first pressure registration).* | Yes No | | 18 (1.3) | |
|  |  | |  | |
